# Aphids and Mycorrhizal Fungi Shape Maternal Effects in *Senecio vulgaris*

**DOI:** 10.3390/plants11162150

**Published:** 2022-08-18

**Authors:** Ruth P. Chitty, Alan C. Gange

**Affiliations:** 1 Tree Health Diagnostic & Advisory Service, Forest Research, Alice Holt Lodge, Farnham, Surrey GU10 4LH, UK; 2 Department of Biological Sciences, Royal Holloway, University of London, Egham, Surrey TW20 0EX, UK

**Keywords:** herbivory, insect, arbuscular mycorrhiza, maternal effects, *Myzus persicae*, *Senecio vulgaris*

## Abstract

Plant performance in any one generation is affected not only by the prevailing environmental conditions, but also by the conditions experienced by the parental generation of those plants. These maternal effects have been recorded in a many plant species, but the influence of external biotic (as opposed to abiotic) factors on shaping maternal effects have been rarely examined. Furthermore, almost all previous studies have taken place over one plant generation, rather than across multiple generations. Here, we studied the influence of insect herbivory and arbuscular mycorrhizal (AM) fungal colonisation on the shaping of maternal effects in the annual forb *Senecio vulgaris.* We grew plants with and without aphids (*Myzus persicae*) and AM fungi (hereafter termed ‘induction events’) over four successive generations, wherein seeds from plants in any one treatment were used to grow plants of the same treatment in the next generation, all in identical environmental conditions. We found strong evidence of maternal effects in the second plant generation, i.e., after one induction event. These plants took longer to germinate, flowered in a shorter time, produced lighter seeds and were shorter and of lower biomass than their parents. Aphid attack tended to enhance these effects, whereas AM fungi had little influence. However, thereafter there was a gradual recovery in these parameters, so that plants experiencing three inductions showed similar life history parameters to those in the original generation. We conclude that experiments investigating maternal effects need to be performed over multiple plant generations and that biotic factors such as insects and mycorrhizas must also be taken into account, along with abiotic factors, such as nutrient and water availability.

## 1. Introduction

Maternal effects occur when the growth conditions experienced by one generation of plants influences the growth of the offspring in the following generation [1]. Such effects can include growth, survival and reproduction of seedlings [2,3,4] as well as production of plant defences against pests and pathogens [5,6,7]. Although ecologically important, these effects are often less commonly observed than within-generation effects, as they can be masked by variation in environmental conditions in the parent and offspring generations [8].

An excellent ‘model plant’ for studying maternal effects is the annual forb *Senecio vulgaris* L. In temperate areas the species shows little dormancy and can germinate at any time of year, giving rise to three or four generations in a year [9]. This species frequently self-pollinates and produces large amounts of viable seed when selfed. Selfing is virtually guaranteed if the non-radiate flower form of the plant is used, with virtually no outcrossing occurring [10]. *S. vulgaris* associates with arbuscular mycorrhizal (AM) fungi and there is some evidence that AM fungal colonisation of maternal plants can increase the reproductive capacity of offspring plants, though this is influenced by nutrient availability [11]. There are very few published studies of maternal effects caused by AM fungi, but in general, offspring seedling vigour is enhanced due to seeds produced on mycorrhizal plants being larger, with higher nutrient contents [12,13].

AM fungi can enhance almost all plant growth and reproductive parameters, including growth rate, leaf number, biomass, flower number and size, seed number, size and viability [14,15]. Given that when maternal plants of *S. vulgaris* were grown in low-nutrient soils, the offspring were smaller, even when grown in a more fertile soil [16], there is clear potential for the beneficial effects of AM fungi on plant growth in the maternal generation to be translated into offspring generations. However, to date, the majority of experiments studying maternal effects in this plant have studied just one offspring generation [11,16,17] and so it is unclear if any such effects propagate or attenuate over successive generations. Understanding these dynamics could be critical in the management of soil seed banks when this species occurs as a weed of crops [18].

It would seem likely that AM-fungal-induced maternal effects would propagate through plant generations, as many of the characteristics influenced by these fungi have been implicated in maternal experiments without the fungi. For example, germination and early seedling growth is often dependent upon materials stored within the seeds [1] with both the plant’s genetical constitution and environmental effects influencing seedling growth [19]. Changes in germination time are influenced by differences in seed dormancy [20,21], but curiously AM fungi can reduce seed viability in soil [22]. Ultimately, changes in seedling growth can lead on to changes in plant development time and the onset of flowering, but whether flowering time is influenced by the maternal environment is unclear. Some species (e.g., *Lolium perenne*) were found to have flowering time influenced by both the plants own genetics and the maternal environment [23], whereas in other species, flowering time was found to be only influenced by the plant’s own genetics [24] or only by the maternal environment [2,25]. It is unknown if AM fungi can exert maternal effects over several generations that lead on to changes in plant development rates and flowering times.

Other important aspects of plant growth, such as final height, leaf number and biomass may also be influenced by maternal effects over one or two generations [26]. In *S. vulgaris*, AM fungal colonisation of parent plants may increase the leaf number of their offspring, but this is dependent on the nutrient status of the parents [11]. It is unknown if certain floral aspects, such as flower number and size can be influenced by maternal effects, though these parameters can be enhanced by AM fungi [15].

In contrast to AM fungi, insect herbivores often have detrimental effects on all the plant growth parameters mentioned above, particularly the critical life history component of seed size [27,28]. Studies of maternal effects involving insect herbivores are relatively few but tend to concentrate on how herbivory in the parental generation may enhance resistance to herbivores in the offspring generation [29,30]. In field situations, it is thought that such changes are adaptive if progeny suffer similar levels of insect attack to those of the previous generation [31]. The defensive phenotype is partly determined by the maternal environment and this phenotype can alter seedling growth [5]. For example, seedling biomass of *Raphanus raphanistrum* was increased in plants whose parents had experienced insect herbivory by larvae of the butterfly *Pieris rapae*. Meanwhile, the larval weight of *P. rapae* was reduced when the larvae fed upon progeny seedlings whose parents had been attacked [5].

Parental effects can affect multiple phenotypes at the same time, so it is interesting that parental effects caused by insect herbivory seemed to override any other effects being passed on [32]. For example, without insect attack, *Verbascum thapsus* seeds had a higher percentage germination when produced by plants in a warmer climate than seeds produced by plants grown in a colder climate. With insect herbivory, the difference in percentage germination between the climates disappeared [32]. However, as with AM fungi, virtually all experiments involving insect herbivory and maternal effects have studied just one or two offspring generations, though see [29] for an exception. Furthermore, no previous work has considered the combined influence of AM fungi and insect herbivory on maternal effects in any species of plant.

In this study, our objective was to determine if AM fungi and an insect herbivore (the aphid, *Myzus persicae*) act or interact to influence maternal effects in *S. vulgaris* across four plant generations. We have already reported on the effects seen in aphid growth and reproduction and the effects of the insect on mycorrhizal colonisation [33], and the experimental design is described fully in that paper. Here, we describe the effects on various vegetative and reproductive parameters of the plant. We hypothesised that AM fungi would have a positive effect on seed size, leading to enhanced seedling vigour and plant growth rates. We also hypothesised that in the absence of AM fungi, insect herbivory in the parental generation may also increase offspring seedling vigour, but that interactions might be seen between the fungi and the insect, given that herbivory is known to reduce AM fungal colonisation, but mycorrhizal presence can increase aphid growth [34,35]. Finally, we hypothesised that any effects observed may not be constant across plant generations, mirroring those seen in the aphid and the mycorrhizal association [33].

## 2. Results

**Figure 1:**
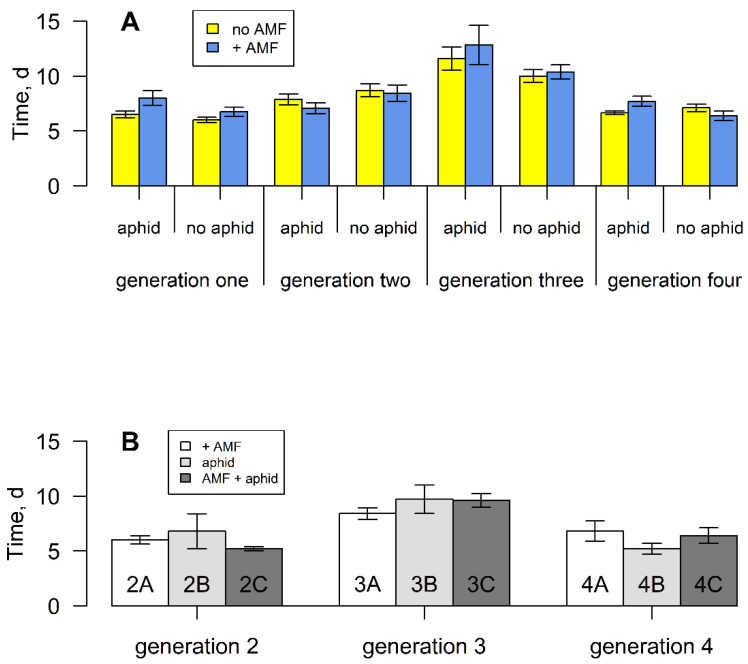
(**A**) Mean seed germination time of *S. vulgaris* grown with or without arbuscular mycorrhizal fungi and aphids for four successive generations. Vertical lines represent ± one standard error. (**B**) Mean seed germination time of *S. vulgaris* grown without AM fungi, aphids or both, from parents that experienced these treatments (2A, B and C; 3A, B and C and 4A, B and C plants in Figure S1).

Overall, the time taken by seeds to germinate differed across inductions (F_3,289_ = 15.984, *p* < 0.001), wherein it increased between zero and two inductions, but decreased after the third induction event in generation four (Figure 1A). Seeds from plants experiencing two inductions (third generation) took the longest time to germinate, but there was a significant interaction between aphids and induction event, with seeds from aphid-attacked parents taking much longer to germinate after two inductions (F_3,289_ = 6.36, *p* < 0.001). Mycorrhizal fungi had no effect on seed germination time overall, but seeds from plants in generation two (after one induction) took longer to germinate than those from the same parents but without the fungi (2A plants in Figure S1) (F_1,39_ = 4.36, *p* < 0.05). A similar recovery was seen in the aphid treatment, where seeds from aphid-free plants (2B in Figure S1) germinated more quickly than those continuously attacked (F_1,39_ = 14.23, *p* < 0.001) (Figure 1). No such differences were seen after two or three inductions.

**Figure 2:**
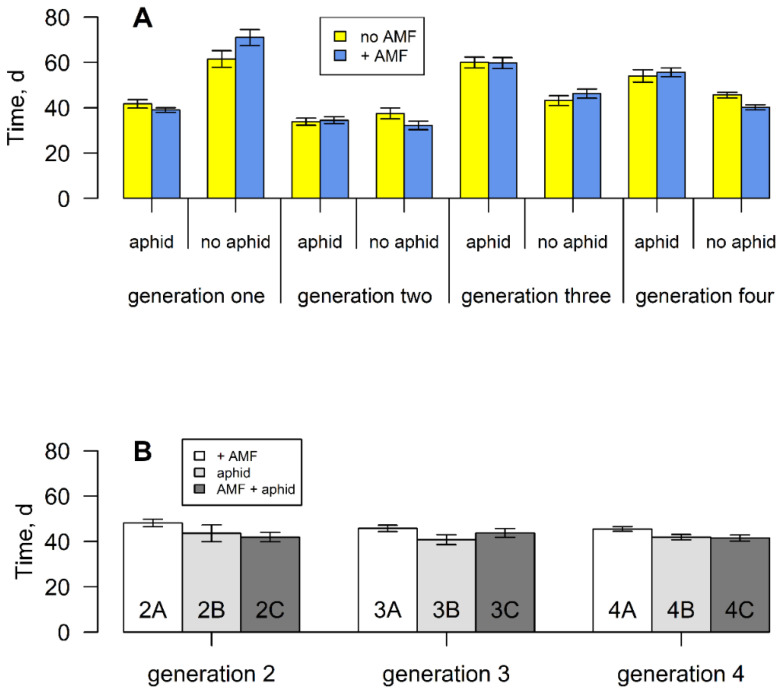
(**A**) Mean time to first flowering of *S. vulgaris* grown with or without arbuscular mycorrhizal fungi and aphids for four successive generations. Vertical lines represent ± one standard error. (**B**) Mean time to first flowering of *S. vulgaris* grown without AM fungi, aphids or both, from parents that experienced these treatments (2A, B and C; 3A, B and C and 4A, B and C plants in Figure S1).

Plants grown from parents that experienced one induction event took a shorter time to produce flowers than those that experienced no, two or three induction events (F_3,289_ = 6.138, *p* < 0.001). Across all induction events, the presence of aphids decreased the time taken to flower (F_1,289_ = 7.063, *p* < 0.01). However, there was a significant interaction term (F_3,289_ = 58.338, *p* < 0.001) between aphids and induction event, as the aphid-induced decrease only happened in generations one and two (after no or one induction). Thereafter, aphid presence increased flowering time (Figure 2A). Interestingly, when aphid presence was removed (3B and 4B plants in Figure S1), flowering time was decreased compared with those plants where it persisted (Figure 2B). Mycorrhizal colonisation had no overall effect on flowering time, but as with seed germination, plants from mycorrhizal parents in generation two without the fungi (2A in Figure S1) took longer to flower than those colonised by the fungi (F_1,39_ = 12.06, *p* < 0.001). No such differences were found in plants experiencing two or three inductions.

**Figure 3:**
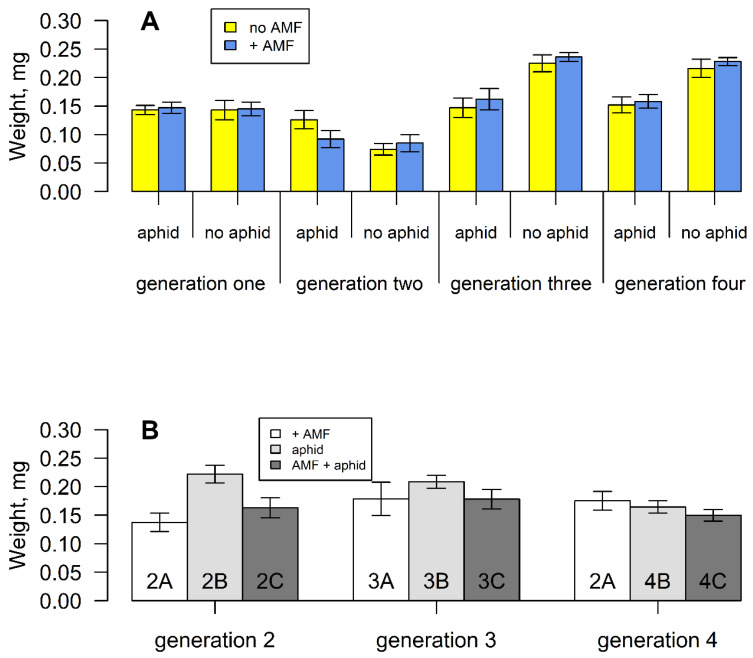
(**A**) Mean seed weight of *S. vulgaris* grown with or without arbuscular mycorrhizal fungi and aphids for four successive generations. Vertical lines represent ± one standard error. (**B**) Mean seed weight of *S. vulgaris* grown without AM fungi, aphids or both, from parents that experienced these treatments (2A, B and C; 3A, B and C and 4A, B and C plants in Figure S1).

Overall, generation two plants (one induction) produced the lightest seeds compared with the other generations, (F_3,289_ = 24.144, *p* < 0.001) (Figure 3A). Across generations, aphid presence had no effect on seed weight, but the effect of aphids differed between inductions, shown by a significant interaction term (F_3,289_ = 3.491, *p* < 0.05). This was because aphid presence increased seed weight after one induction (when seeds were lightest) but decreased it after two and three inductions (Figure 3A). When the aphid presence was removed after one induction (2B plants in Figure S1), seeds remained large (Figure 3B). However, this effect waned after two and three inductions (3B and 4B plants in Figure S1). Mycorrhizal fungi had no effect on seed weight across all generations.

**Figure 4:**
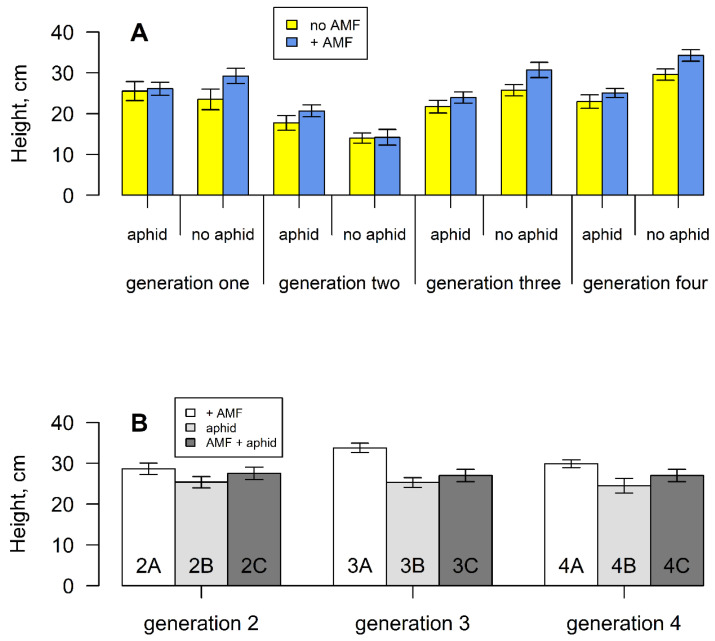
(**A**) Mean final plant height of *S. vulgaris* grown with or without arbuscular mycorrhizal fungi and aphids for four successive generations. Vertical lines represent ± one standard error. (**B**) Mean final plant height of *S. vulgaris* grown without AM fungi, aphids or both, from parents that experienced these treatments (2A, B and C; 3A, B and C and 4A, B and C plants in Figure S1).

Plants following one induction event were shorter than those in any other generation (F_3,298_ = 23.171, *p* < 0.001) (Figure 4A). Overall, aphids had no effect on plant height, but attacked plants following one induction were taller than non-attacked plants. If aphids were removed (2B plants in Figure S1), plants were shorter (Figure 4B). In all other generations the aphid-attacked plants were shorter than the non-attacked plants, leading to a significant interaction term (F_3,298_ = 3.353, *p* < 0.05). However, if aphids were removed, (3B and 4B plants in Figure S1), plants were larger (Figure 4B). Irrespective of aphid presence, AM fungi increased final height consistently across plant generations. (F_3,298_ = 4.48, *p* < 0.05). When plants were then grown without the fungi (2A, 3A and 4A in Figure S1), the size difference remained (Figure 4B).

**Figure 5:**
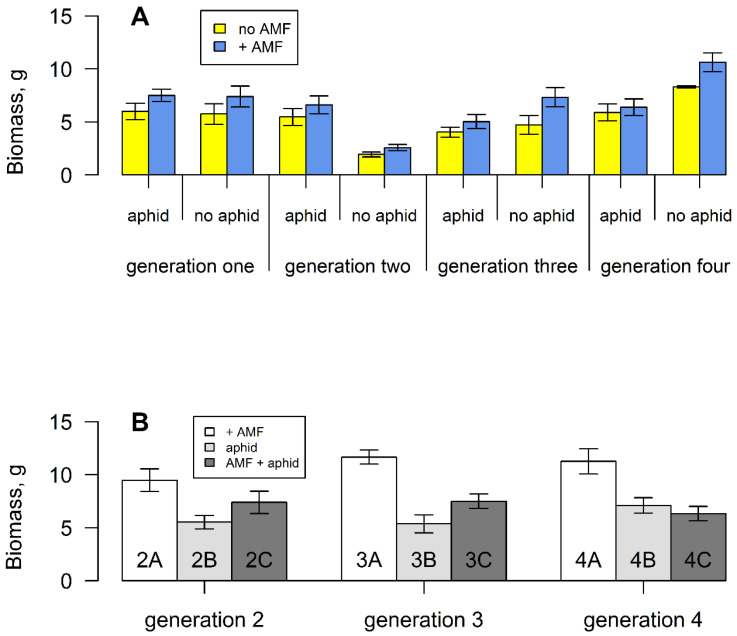
(**A**) Mean total dry biomass of *S. vulgaris* grown with or without arbuscular mycorrhizal fungi and aphids for four successive generations. Vertical lines represent ± one standard error. (**B**) Mean total dry biomass of *S. vulgaris* grown without AM fungi, aphids or both, from parents that experienced these treatments (2A, B and C; 3A, B and C and 4A, B and C plants in Figure S1).

The reduction in height in generation two (one induction) translated into a reduction in final biomass (F_3,298_ = 15.737, *p* < 0.001) (Figure 5A). However, this only occurred when aphids were absent, leading to a highly significant interaction term between inductions and aphids (F_3,298_ = 11.67, *p* < 0.001). When aphids were removed (2B, 3B and 4B plants in Figure S1), plants tended to be larger than those suffering continuous attack (Figure 5B). Over all induction events, AM-fungal-colonised plants had higher biomass than non-colonised plants (F_1,298_ = 4.376, *p* < 0.05), with no interactions between mycorrhizal fungi and inductions or aphids. However, when plants were subsequently grown without AM fungi (2A, 3A and 4A in Figure S1), the enhancement of biomass remained (Figure 5B).

## 3. Discussion

We found significant maternal effects across a variety of growth and reproductive parameters in *S. vulgaris*. These effects were most clearly seen after one induction (generation two plants) with seeds taking longer to germinate and plants flowering in a shorter time, while being shorter in stature, lighter, and producing smaller seeds. Across generations, there were few clear overall effects of AM fungi or aphids on these parameters, providing little support for our first two hypotheses. However, maternal effects and those involving fungi and insects were inconsistent across generations, proving strong support for our third hypothesis. In particular, the effects of aphids were dependent on induction number, producing many significant interaction terms. Furthermore, the transient and inconsistent nature of the maternal effects were clearly seen when plants were relieved of the treatments and grown without aphids or mycorrhizal fungi. In many cases, these plants showed different growth or reproductive parameters to their conspecifics experiencing continued herbivory or colonisation, and were more similar to the control (insect and mycorrhiza free) plants.

Mycorrhizal colonisation was around 10–15% root length colonised and was highest in generations one and four [33] but seemed to have little influence on maternal effects. This could be due to a lack of abiotic stress experienced by the plants. Mycorrhizal fungi have developed mechanisms to deal with stress experienced by the host plant [36]. As our plants were not subjected to environmental stress, mycorrhizal mechanisms may not have been deployed in this experiment. The inconsistent effects were exemplified in the time that seeds from each generation took to germinate. Germination time increased between inductions zero and two but decreased again after induction event three, with the increase largely due to herbivory. The change in time taken to germinate may be due to epigenetic changes in genes regulating seed dormancy through the deacetylation and methylation of DNA [37]. These epigenetic changes can remove or put into place the inhibiting factors that prevent or slow germination [37,38]. The environment was kept the same for each generation, so the plant could be passing on information for a better survival in the same environment [39]. Insect herbivory has been shown to decrease or increase seed viability across plant generations, dependent upon the plant species identity [40]. However, such experiments have not taken place over multiple generations, and our results show the value of such multi-generational studies. Germination time is linked to seed dormancy, and it could be that the maternal plants are able to increase seed dormancy so the seeds do not germinate in the same environment immediately [41]. Third-generation seeds may have had a longer germination time as the maternal effects on seed resources began to accumulate. Insect herbivory has been linked to less resources being placed into the seed [27], so maternal plants that are less affected by insect herbivory are likely to be better equipped to increase resource allocation to the seeds and these seedlings may germinate faster [27]. In the case of *S. vulgaris,* it may not be the absolute size of seeds that is important, but their quality, in terms of nutrient reserves. In this experiment, seeds from aphid-attacked plants after one or two inductions contained lower levels of nitrogen [33], perhaps indicating that their nutrient reserves were lower, which could lead to impaired germination. In contrast, when seeds were taken from mycorrhizal parents, and plants then grown without the fungi, they were larger than those from non-mycorrhizal parents. This suggests a positive effect on seed reserves, and hence seedling and plant vigour. This provides partial support for our first hypothesis, which was based upon AM fungal increases in seed size. Rather, it appears that seed quality is the key factor by which AM fungi exert some maternal effects, most likely through enhanced phosphate status [12,13].

Seeds produced by mycorrhizal plants often show enhanced viability and germination [15], though the effects are often dependent on environmental conditions in the offspring generation [11,16]. Plants in this experiment were grown in constant, benign environmental conditions, with adequate nutrients and water. Although logistically challenging, it would be interesting to examine the role of both aphids and AM fungi on maternal effects in conditions where nutrients and/or water are limiting. As *S. vulgaris* can have three or four generations per year, it is certain that each generation will experience differences in temperatures and water availability [9]. Therefore, we suggest that experiments involving maternal effects need to be conducted where a range of biotic and abiotic factors are manipulated. We would expect the results to show that both within-generation and maternal effects play a role in plant performance, as seen with other field experiments over one induction event [42]. Furthermore, maternal effects may only be visible depending on the abiotic or biotic factors manipulated [17].

Differences in seed size can lead to differences in development time, and ultimately the development of size hierarchies in plant populations [43,44]. We measured flowering time as our development parameter, which was shorter after zero or one inductions, when aphids were present. However, after further inductions, as seed germination increased, so did the time taken to flower.

Flowering is important for reproductive success, so the time taken to flower is important for a rapid-cycling annual such as *S. vulgaris*. It was assumed that plants would speed up flowering time throughout all induction events to increase reproduction, but this did not occur. However, flowering has been explored with epigenetic changes to chromatin [45]. A delay in flowering time was found to be an epi-mutant of the flowering Wageningen (*FWA*) gene in *Arabidopsis thaliana* [46], which led to prevention or delay of proper flowering. This suggests that the *S. vulgaris* plants after induction event two may have experienced epigenetic changes, especially to the *FWA* gene, as flowering was delayed, but only when aphids were present. Insect herbivory has often been shown to cause a decrease in time taken to flower [47], but this was not the case here, as it was dependent upon induction event. Given that when aphids were removed, the subsequent plants flowered more rapidly than those after two or three inductions suggests that the effects here persist over generations and are likely caused by epigenetic changes or long-term resource allocation patterns to the seeds.

Seeds produced by plants from generation two (one induction) were considerably lighter than those from any other generation, but the effects did not appear to be cumulative. Variation in seed size can result in progeny that can effectively cope with unpredictable but recurrent instabilities in their environment [48]. The variation in *S. vulgaris* seed weight could help the plant to cope with spatial and temporal variation in insect attack and/or mycorrhizal colonisation. Similar to other studies that took place over two generations, we found that insect attack increased seed size [40]. However, after three or four generations, this effect was reversed, again showing the importance of multi-generational studies of this type. The nutrient content of *S. vulgaris* seeds followed the same pattern as seed weight [33], suggesting that there was an increase in maternal nutrition. The fact that when aphids were removed the seeds were similar in weight to those of controls suggests that the aphid influence on maternal effects was a transient one. Meanwhile, mycorrhizal colonisation did not affect seed weight, which agrees with previous studies [11].

After one induction event, both the final plant height and biomass were reduced but both subsequently recovered. There was a clear effect of AM fungal colonisation on these parameters with both being increased by mycorrhizal presence. Contrary to expectations however, aphid feeding did not reduce height or biomass but instead increased these measurements after one induction. Furthermore, these increases occurred irrespective of mycorrhizal presence. Mycorrhizal colonisation often (but not always) increases aphid performance [49,50], but in this study, colonisation had no effect on aphid growth [33]. The mechanism of AM fungal effects on aphids is thought to be due to the fungi increasing the ease of feeding for aphids through vascular bundle size [51], but it is possible that this does not occur in this plant. It is unclear why aphid feeding increased plant growth, but such an effect has been reported before [52]. In that study, herbivory in the maternal generation of *Populus* spp. caused an increase in plant biomass in the next generation, but there was a trade-off between maintenance of offspring growth and phytochemical defences. In the *Populus* spp. it was found that the ‘decision’ to increase growth at the expense of defence was taken [52]. It could be that *S. vulgaris* put more resources into growth and decreased the amount of resources put into chemical defence. Meanwhile, over three generations of *Polygonum persicaria,* the offspring of stressed individuals had a larger biomass than the offspring of non-stressed plants [26] and the effects were found to be cumulative over two generations. This could be the case with *S. vulgaris*, as the increase in dry biomass on aphid-attacked plants occurred in the second and third induction events, so the effects could be cumulative. However, one study linked the presence of a specific endophyte (*Epichloë gansuensis*) and maternal effects to increases in plant height in a grass [53]. It may be that there is a similar endophyte present within *S. vulgaris* that affects aphid and plant growth over multiple generations, especially as endophytes have been shown to have vertical transmission in this species [54]. Furthermore, several of the endophyte species that occur within *S. vulgaris* can have positive effects on aphid and plant growth [54,55]. However, it has recently been shown that the effects of AM fungi on insects depend on endophyte presence (and vice versa) [55], and so endophytes are a further biotic factor that should be considered in studies of maternal effects.

## 4. Materials and Methods

### 4.1. Plant Growth Conditions

The experimental design is described fully in Chitty and Gange [33] and shown in Figure S1. The mycorrhizal colonisation results from the plant described here were previously reported in [33], as this was a large, long-term experiment with multiple parameters measured. Briefly, seeds of the non-radiate form of *S. vulgaris* were collected from a wild population and grown for one generation in controlled conditions (20 °C, 78% RH and 16 h daylight) to minimise any influence of the parental environment [56]. Seeds were collected from these plants and thereafter plants were grown for four generations, in which there were four treatments: control (no mycorrhizal fungi or insects), addition of a mixed inoculum of AM fungi (‘Rootgrow’ (PlantWorks Ltd., Sittingbourne, Kent, UK), containing *Claroideoglomus claroideum, Funneliformis geosporus, F. mosseae, Glomus microaggregatum*, and *Rhizophagus irregularis*), infestation by aphids (*M. persicae*) or both AM fungi and aphids. Seeds from any one treatment were used to start that same treatment in the next generation in the main experiment. In addition, seeds from aphid only, mycorrhiza only and aphid + mycorrhizal plants were taken from generations one, two and three and offspring grown without insects or fungi. These are depicted in Figure S1 as 2A, 2B, 2C, 3A, 3B, 3C and 4A, 4B and 4C plants. In all cases there were 20 replicates of each treatment. Full details of the soil and growth conditions are given in [33].

### 4.2. Plant Growth and Reproduction Measurements

The plant parameters for each of the four generations reported here are germination time of seeds, time to first flowering, mean seed weight, final plant height and biomass. To measure seed germination, a random sample of 20 seeds was collected from each mature plant and placed on moist filter paper in a petri dish. The time (in d) to first visible growth (radicle emergence) was recorded for each seed and a mean taken per plant. Time to first flowering was recorded as the difference (in d) between seedling emergence and the opening of the first flower bud on each mature plant. To record seed weight, a random sample of 20 seeds was taken from each plant and seeds weighed individually on a microbalance to obtain a mean value per plant. Height in cm was measured with digital callipers from the soil to the top of the highest flower. When other measurements had been taken, each plant was severed at soil level and the vegetative biomass dried at 60 °C for 48h, prior to weighing. As each experimental run involved successive insect attack events or colonisation by AM fungi, we refer to these as ‘inductions’, for consistency with our previous paper and others of similar design [26,30]. Overall, there were three induction events over the four plant generations, as shown in Figure S1.

### 4.3. Statistical Analysis

All analyses were performed in R 4.0.5 [57]. Normality tests were performed on whole data sets and data were transformed, if necessary, using lambda calculated by the Box–Cox transformation [30].

Data for germination time, time to first flower, seed weight, plant height and final biomass were subjected to three-factor ANOVA, employing aphid absence/presence, AM fungi absence/presence and induction event as the main effects. In each case, the minimal adequate model was considered, following stepwise deletion of non-significant interaction terms [33].

We also used one factor ANOVA to examine whether cessation of any treatment caused differences in plant parameters compared with successive inductions. Thus, for example, for each plant parameter, AM-fungi-only plants grown in generation two (i.e., after one induction) were compared with those from the same parents, but lacking the fungi (2A plants in Figure S1). Meanwhile, aphid-only plants in generation two were compared with those from the same parents but without aphids (2B plants in Figure S1), etc.

## 5. Conclusions

Maternal effects in *S. vulgaris* were apparent in seed germination time, flowering time, seed weight, plant height and final biomass. Mycorrhizal fungi had little effect on these parameters, but aphid attack increased or decreased them, depending upon the number of generations of plants experiencing herbivory. This study shows the value of conducting experiments on maternal effects on plants across multiple generations. In future, such experiments need to be conducted while simultaneously manipulating abiotic environmental conditions such as soil nutrient levels and water status and biotic factors such as herbivory and beneficial fungi. These experiments will be challenging but are needed in order to fully understand how biotic and abiotic factors shape maternal effects in field populations.

## Data Availability

All data belonging to this manuscript are available from the corresponding author upon reasonable request.

## Supplementary Materials

The following supporting information can be downloaded at: https://www.mdpi.com/article/10.3390/plants11162150/s1, Figure S1: Diagram showing experimental treatment set up. Aphid picture represents plants that experienced insect herbivory. Mycorrhizal slide photograph represents plants that experienced mycorrhizal colonisation.
