# Supplementary figures and images for: Aphids and Mycorrhizal Fungi Shape Maternal Effects in Senecio vulgaris
Source: Plants (Basel). 2022 Aug 18;11(16):2150. doi: 10.3390/plants11162150 (PMC9415133; doi:10.3390/plants11162150)

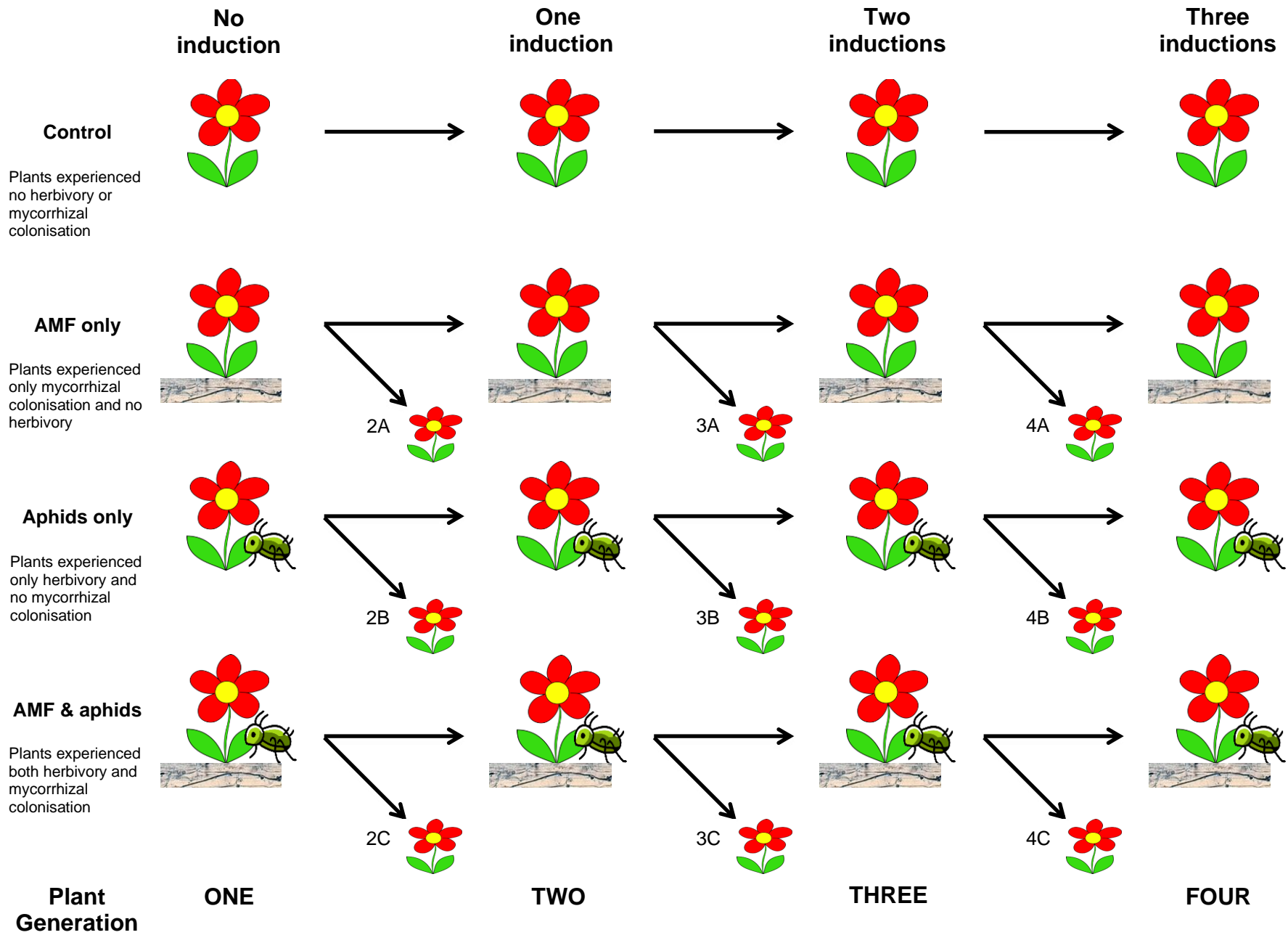

Supplement: Supplementary file 1 [file plants-11-02150-s001.zip › Supplementary Figure S1.pdf]
